# ITGBL1 is a new immunomodulator that favors development of melanoma tumors by inhibiting natural killer cells cytotoxicity

**DOI:** 10.1186/s12943-020-01306-2

**Published:** 2021-01-07

**Authors:** Yann Cheli, Meri K. Tulic, Najla El Hachem, Nicolas Nottet, Arnaud Jacquel, Maeva Gesson, Thomas Strub, Karine Bille, Alexandra Picard-Gauci, Henri Montaudié, Guillaume E. Beranger, Thierry Passeron, Pierre Close, Corine Bertolotto, Robert Ballotti

**Affiliations:** 1 Université Nice Côte d’Azur, INSERM U1065, Team1 Biology and pathologies of melanocytes. Equipe labellisée ARC 2019, 06000 Nice, France; 2 Université Nice Côte d’Azur, INSERM, U1065, Team12 Study of the melanocytic differentiation applied to vitiligo and melanoma, 06000 Nice, France; 3 Laboratory of Cancer Signaling, University of Liège, Liège, Belgium; 4 Université Nice Côte d’Azur, INSERM, U1065, Team2 Cell death, differentiation and cancer, 06000 Nice, France; 5 Université Nice Côte d’Azur, INSERM, U1065, Imaging platform, 06000 Nice, France; 6 CHU NICE, Département de Dermatologie, 06000 Nice, France

**Keywords:** Melanoma, ITGBL1, MITF

## Abstract

Resistances to immunotherapies remains a major hurdle towards a cure for melanoma in numerous patients. An increase in the mesenchymal phenotype and a loss of differentiation have been clearly associated with resistance to targeted therapies. Similar phenotypes have been more recently also linked to resistance to immune checkpoint therapies. We demonstrated here that the loss of MIcrophthalmia associated Transcription Factor (MITF), a pivotal player in melanocyte differentiation, favors the escape of melanoma cells from the immune system. We identified Integrin beta-like protein 1 (ITGBL1), a secreted protein, upregulated in anti-PD1 resistant patients and in MITF_low_ melanoma cells, as the key immunomodulator. ITGBL1 inhibited immune cell cytotoxicity against melanoma cells by inhibiting NK cells cytotoxicity and counteracting beneficial effects of anti-PD1 treatment, both in vitro and in vivo*.* Mechanistically, MITF inhibited RUNX2, an activator of ITGBL1 transcription. Interestingly, VitaminD3, an inhibitor of RUNX2, improved melanoma cells to death by immune cells. In conclusion, our data suggest that inhibition of ITGBL1 might improve melanoma response to immunotherapies.

**Supplementary Information:**

The online version contains supplementary material available at 10.1186/s12943-020-01306-2.

Despite recent therapeutic improvements, the prognosis of patients with metastatic melanoma is still very pejorative. Targeted therapies (TT) using BRAF in combination with MEK inhibitors, have shown very high response rates. However, quasi systematic acquired resistances have limited the improvement of patient survival [1]. Immuno-therapeutic approaches targeting negative immune check points (ICT) brought stunning improvement in patient survival. However, most patients are resistant or develop resistance to ICT, highlighting the need of new complementary therapeutic approaches to overcome these resistances.

Genetic events, including mutations that cause resistance to TT or ICT have been extensively described. However, the main cause of resistance to TT is non-genetic. It implies a rewiring of the transcriptional program allowing the adaptation of melanoma cells to stressful conditions imposed by the micro-environment or by the treatment itself. Despite the diversity of non-genetic mechanisms of resistance, loss of MITF, loss of differentiation, as well as implementation of a pseudo-EMT and inflammatory phenotype [2] are central to resistance to TT [3]. More recently, such de-differentiated profile has been also associated with resistance to ICT [4].

## MITF inhibition decreases the cytotoxicity of immune cells through the secretion of ITGBL1

**Fig. 1:**
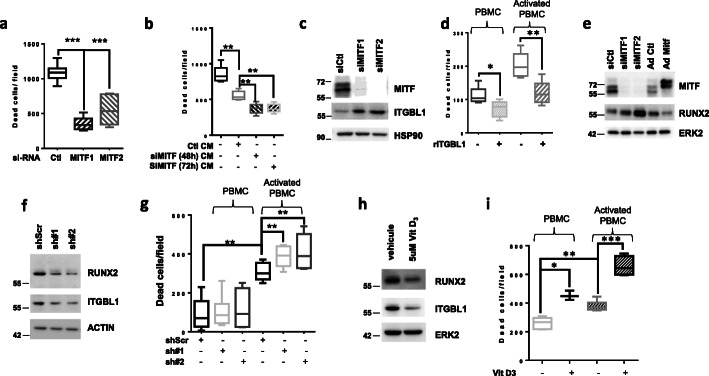
MITF expression modulates immune system response through a soluble, secreted factor ITGBL1 via RUNX2. **a** Melanoma cells were transfected with siRNA control or 2 different siRNA directed against MITF. Forty-eight hours later, activated PBMCs were added to cells and acquisition using Incucyte was performed. Quantification of melanoma cells death is displayed for each condition. **b** Activated PBMCs were incubated for 48 h in conditioned media from siCtl or siMITF melanoma cells and subsequently incubated with naïve 501Mel melanoma cells. Quantification of melanoma cell death after incubation with PBMCs is shown. All graphs represent mean+/−SD of 3 independent experiments. **c** 501Mel were transfected with two different siRNA for MITF (A) for 48 h. Protein lysates were separated by SDS page and blotted for MITF and ITGBL1 expression. HSP90 was used as a loading control. **d** Resting or activated PBMCs were incubated for 48 h in presence or absence of recombinant ITGBL1 (5 ng/ml). PBMCs were subsequently added to 501Mel melanoma cells and cell death was analyzed with Incucyte. Quantification of melanoma cell death is displayed as the mean+/−SD of 3 independent experiments. **e** WM3912 melanoma cells were transfected with control or MITF siRNA or infected with control or MITF adenoviruses. Proteins were probed for MITF and RUNX2 proteins expression. ERK2 was used as loading control. **f** 501Mel cells were infected with 2 different RUNX2 shRNA (sh#1, sh#2) encoding lentiviruses or its control shRNA (shScr). Proteins were probed for MITF and RUNX2 expression. ERK2 was used as a loading control. **g** 501Mel cell death was monitored with Incucyte experiments in presence of resting or activated PBMCs. Quantification of melanoma cell death is displayed as the mean+/−SD of 3 independent experiments. **h** WM9 melanoma cells were treated with 5 μM of VitD3 for 24 h, then RUNX2 and MITF proteins analyzed by western blot. ERK2 was used as loading control. **i** WM9 melanoma pretreated with 5 μM VitD3 were incubated with resting or activated PBMCs. Melanoma cell death was quantified using Incucyte. Results show as mean+/−SD of one representative experiment done in triplicate

MITF silencing with 2 different MITF siRNA, caused a 2-fold decrease in 501Mel cells death induced by activated PBMCs (Fig. 1a). These effects can be ascribed either to the inhibition of the intrinsic ability of melanoma cells to be killed by immune cells, or to decreased cytotoxic function of immune cells mediated by the secretion of immunomodulating agents. When PBMCs were first incubated with conditioned medium (CM) from siCtl or siMITF treated 501Mel, we observed that CM from siMITF transfected cells significantly decreased the cytotoxicity of PBMCs on untreated melanoma cells (Fig. 1b). This result indicates that melanoma cells secrete negative immunomodulating agents whose secretion is increased upon MITF silencing. MITF low cells are known to have a pro-inflammatory secretory profile characterized by the production of numerous cytokines and immune regulators. To identify key secreted factors that might impact the immune system, we integrated the transcriptomic profile of melanoma cell lines (CCLE Broad) expressing low MITF versus high MITF with the genes up regulated in non-responder to immune therapies [5]. We identified 40 genes that are up regulated in both conditions (sup. fig. 1A). Among these genes, 17 were described to encode secreted proteins that might affect the capacity of immune cells to kill melanoma cells (sup. fig. 1B).

Among these proteins, ITGBL1 was previously reported to be a bad prognosis factor in several cancer such as gastric, ovarian, lung and colorectal cancers [6–9]. First, we demonstrated that MITF silencing in 501Mel cells increased ITGBL1 expression (Fig. 1c) confirming that ITGBL1 was negatively modulated by MITF. This was also observed in WM3912 cells (sup. fig. 1C). The key role of secreted ITGBL1 in the inhibition of immune cell cytotoxicity was demonstrated by showing that the CM from SKMEL28 over expressing ITGBL1 decreased the cytotoxicity of PBMCs towards naive 501Mel melanoma cells. The cytotoxicity of PBMCs was rescued by immuno-depletion of ITGBL1 (sup. fig. 1D, E). Next, we showed that treatment of activated PBMCs with recombinant ITGBL1 decreased their ability to kill 501Mel melanoma cells (Fig. 1d). Furthermore, increased GZMB and IFNγ mRNA expression upon activation of PBMCs with PMA/ionomycin was abrogated by rITBL1 (sup. fig. 2A). Taken together these observations support an inhibition of immune cell activation by rITGBL1.

To gain insight on the mechanism by which MITF regulated ITGBL1, we focused our attention on RUNX2 that was reported to regulate ITGBL1 expression in breast cancer cells [10]. Analysis of the TCGA melanoma database revealed a significant increase in ITGBL1 expression in tumors with high RUNX2 expression, whereas MITF was decreased in these same patients (sup. fig. 2B) suggesting that in melanoma cells, ITGBL1 is positively regulated by RUNX2 and negatively regulated by MITF. Next, the epistatic interaction between MITF and RUNX2 was demonstrated using siRNA approach to silence MITF or adenoviral overexpression of MITF in WM3912 (Fig. 1e) or in 501Mel cells (sup. fig. 2C, D). In both cell lines, while inhibition of MITF led to an increase in RUNX2, MITF overexpression lead to a dramatic decrease in RUNX2 expression at protein and mRNA level. The binding of MITF in the vicinity of RUNX2 gene as confirmed by analysis of public MITF ChIP-Seq data [11] (sup. fig. 2E).

Next, we showed that ITGBL1 expression was dampen upon RUNX2 silencing in 501Mel melanoma cells (sh#1, sh#2) compare to control (shScr) (Fig. 1f). In the same conditions, RUNX2 silencing increased melanoma cell death mediated by PBMCs compared to control cells (Fig. 1g).

Finally, we evaluated the effect of the treatment of melanoma cells with Vit D3, which has been shown to inhibit RUNX2 expression [12], on the expression of ITGBL1 and on the PBMCs cytotoxic activity. For this purpose, we pretreated WM9 or 501Mel melanoma cells with VitD3 (Fig. 1h, sup. fig. 2f) and confirmed the decrease in ITGBL1 and RUNX2. Exposure of PBMCs to vitamin D3-treated melanoma cells increased cytotoxic activity of PBMCs in both resting and activated PBMCs (Fig. 1i, sup. fig. 2f). Together, these data thus suggest that Vit D3 might potentiate immune-mediated melanoma cell death by repressing RUNX2 and ITGBL1 expression.

## ITGBL1 inhibits NK cells cytotoxicity towards melanoma cells

**Fig. 2:**
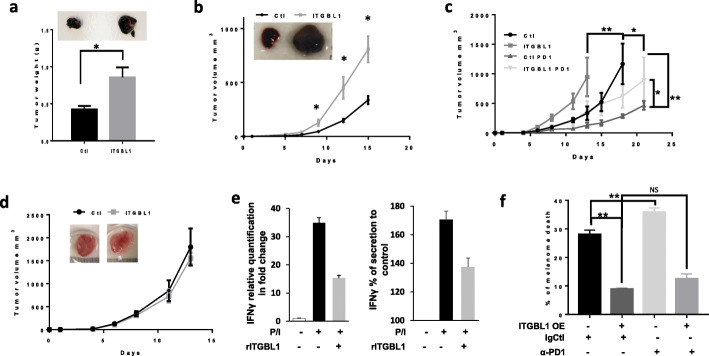
ITGBL1 modulates tumor growth by inhibiting natural killer. **a** 0.15 × 10^6^ B16F10 cells overexpressing or not ITGBL1 were injected subcutaneously in C57BL/6 J, and tumor weight was monitored after 12 days (mean tumor weight in g ± s.e.m.). Picture of representative tumors at 12 days is shown on the top (*n* = 6 mice, *P < 0.05). B) mRNA form tumor overexpressing ITGBL1 (ITGBL1) or not (Ctl) were quantified for IFNγ and GZMB by QPCR and expressed as relative quantification in fold change. **b** 0.15 × 10^6^ B16F10 cells overexpressing or not ITGBL1 were injected subcutaneously in Nude mice and tumor volume was monitored for 15 days (mean tumor volume in g ± s.e.m.). Picture of representative tumors at 15 days is shown on the top (*n* = 6 mice, *P < 0.05). **c** 0.15 × 10^6^ BP cells overexpressing (ITGBL1) or not ITGBL1(Ctl) were injected subcutaneously in C57BL/6 J. When tumor reached 50mm^3^, mice were treated with anti-PD1 (PD1) and tumor growth was monitored. Tumor volume is displayed (mean tumor volume in mm^3^ ± s.e.m.). (*n* = 6 mice, *P < 0.05). **d** 0.15 × 10^6^ BP cells overexpressing or not ITGBL1 were injected subcutaneously in NSG, and tumor growth was monitored for 14 days (mean tumor volume in mm^3^ ± s.e.m.). Picture of representative tumors at 14 days is shown on the top (*n* = 6 mice). **e** NK-92 cells were activated (PMA/iono) for 1 h in presence or absence of ITGBL1 (10 ng/ml). mRNA was extracted and analyzed by QPCR for IFNγ expression. Results shown are mean+/−SD of 3 independent experiments (left panel). IFNγ secretion in activated NK-92 cells incubated for 6 h with or without ITGBL1. Results shown are mean+/−SD (right panel). **f** Red stained Skmel-28 cells, overexpressing ITGBL1 or not, were cocultured with NK at ratio 1 NK/1 melanoma and treated with anti-PD1 (5 μg/ml) or its isotype control for 24 h. Cells were analyzed by FACS with dapi and % of melanoma positive for dapi was quantified and expressed as % of melanoma death (mean+/−SD, *n* = 3, *P < 0.05, **P < 0.001)

To confirm ITGBL1 effect in vivo, we engineered B16F10 cells over-expressing ITGBL1. Subcutaneous injection of parental of ITGBL1 overexpressing B16F10 in C57BL/6 J mice showed a 2-fold increase in the weight of tumors with ITGBL1 overexpressing B16 cells compared to parental cells (Fig. 2a). QPCR assays indicated that ITGBL1 overexpressing tumors had a decrease in IFNγ and GZMB mRNA level (sup. fig. 3A). Then, we performed similar experiments using athymic nude mice that lack mature functional T cells but have more NK cells [13]. Tumors from B16F10 cells overexpressing ITGBL1 developed faster compared to B16F10 control cells (Fig. 2b). With ITGBL1 overexpressing B16 cells, tumor volume at day fifteen presented a three-fold increase compared to parental cells and showed a decrease in IFNγ and GZMB mRNA (sup. fig. 3B) confirming decreased immune cell activity. These results indicated that ITGBL1 did not act on immune T-cells.

To confirm these results, we performed similar experiment with of BP melanoma cells that are much more immunogenic than B16 cells and tested the effect of ITGBL1 in the context of anti-PD1. As expected, treatment with anti-PD1, decreased parental BP tumor growth compared to control Ig (Fig. 2c). When using BP overexpressing ITGBL1, tumors developed faster than parental BP tumors. In this case also, anti-PD1 treatment decreased tumor growth, but ITGBL1 overexpressing tumors grew significantly faster than parental tumors submitted to the same treatment, indicating that ITGBL1 dampen the beneficial effect of anti-PD1.

To further evaluate the involvement of the immune system in the effect evoked by ITGBL1 overexpression, we used NSG mice that lack T, B and NK cells. In this case, tumors from control and ITGBL1 overexpressing BP cells had grown at the same rate (Fig. 2d), suggesting that ITGBL1 inhibited mainly the NK cell activity to favor growth of melanoma tumors.

To confirm this hypothesis, we used the NK-92 cell line and showed that rITGBL1 decreased the IFNγ mRNA expression and secretion by two-fold (Fig. 2e) in stimulated NK cells. Using SKMEL28 overexpressing ITGBL1, we observed that ITGBL1 overexpressing cells are less sensitive to cell death by NK-92 with greater than two-fold decrease in cell death in all the conditions (Fig. 2f, sup. fig. 4A). Furthermore, although anti-PD1 treatment slightly increased the cytotoxicity of NK-92, it did not rescue the inhibition evoked by ITGBL1 overexpression. The effect of ITGBL1 on NK cells was further confirmed, using CD56 sorted NK cells from a healthy donor (sup. fig. 4B)

Finally, to confirm that indeed, ITGBL1 is regulated by MITF, we modulated MITF expression in melanoma cells with different genetic background (NRAS mutated, BRAF/NRAS WT) and used melanoma cells with no MITF. These experiments have demonstrated that modulation of MITF regulates RUNX2 and ITGBL1 (sup. fig. 5). Importantly, we have shown that cells expressing low MITF and high ITGBL1 are protected from death following exposure to NK-92 cells whilst loss of ITGBL1, evoked by MITF forced expression, increased cells death by the same NK cells.

We also investigated the role of beta-catenin, an upstream regulator of MITF and RUNX2. In 501Mel cells, known to have a β-catenin activating mutation, we confirmed a high LEF/TCF activity in 501Mel compared to A375. As expected, WNT3a activated the LEF/TCF luciferase reporter in A375 cells, but not in 501Mel cells (sup. fig. 6a). In A375, activation of β-catenin pathway by WNT3a led to increased RUNX2 and ITGBL1 but failed to increase MITF expression. When WNT3a-treated A375 cells were exposed to NK92 cells, we observed a decrease in cell death caused by NK cells (sup. fig. 6b). Therefore, it seems that the WNT pathway could regulates RUNX2, ITGBL1 and susceptibility to NK cells induced death, independently of MITF.

Our results have identified ITGBL1 as a new immunomodulator secreted by melanoma cells. ITGBL1 inhibits immune cell-mediated destruction of melanoma cells through the modulation of tumoral innate immune system and the decrease in NK cell activity. Importantly, studies in immunocompetent mouse model, shows that ITGBL1 impairs the anti-tumor effect of anti-PD1, suggesting that ITGBL1 might be key player in the resistance to ICT. At a mechanistic point of view, MITF represses RUNX2 which is strong transcriptional activator of ITGBL1. Interestingly, vitamin D3, a RUNX2 inhibitor, that have already shown to have a positive effect on the innate immune system [14], decreases ITGBL1 expression and markedly improves immune-mediated death of melanoma cells, encourage future research to evaluate the effect of Vit D3 supplementation during immuno-therapy treatment in patients with melanoma.

## Supplementary Information

**Additional file 1.**

## Data Availability

The dataset used during this study are available from TCGA.

## Abbreviations

ICT: Immune Checkpoint Therapy
TT: Target therapy
EMT: Epithelial Mesenchymal Transition
MITF: Microphthalmia associated transcription factor
PBMCs: Peripheral blood mononuclear cells
CM: Conditioned media
ITGBL1: Integrin beta like protein 1
PD-1: Program cell death protein
ChIP: Chromatin immunoprecipitation
Vit D3: Vitamin D3
RUNX2: Runt-related transcription factor 2
SKCM: Skin cutaneous melanoma dataset
TCGA: The Cancer Genome Atlas
PMA: Phorbol 12-myristate 13-acetate
QPCR: Quantitative Polymerase Chain Reaction
GZMB: Granzyme B
IFNG: Interferon gamma
