# Correction to: ITGBL1 is a new immunomodulator that favors development of melanoma tumors by inhibiting natural killer cells cytotoxicity

**DOI:** 10.1186/s12943-021-01319-5

**Published:** 2021-01-27

**Authors:** Yann Cheli, Meri K. Tulic, Najla El Hachem, Nicolas Nottet, Arnaud Jacquel, Maeva Gesson, Thomas Strub, Karine Bille, Alexandra Picard-Gauci, Henri Montaudié, Guillaume E. Beranger, Thierry Passeron, Pierre Close, Corine Bertolotto, Robert Ballotti

**Affiliations:** 1 Université Nice Côte d’Azur, INSERM U1065, Team1 Biology and pathologies of melanocytes. Equipe labellisée ARC 2019, 06000 Nice, France; 2 Université Nice Côte d’Azur, INSERM, U1065, Team12 Study of the melanocytic differentiation applied to vitiligo and melanoma, 06000 Nice, France; 3 Laboratory of Cancer Signaling, University of Liège, Liège, Belgium; 4 Université Nice Côte d’Azur, INSERM, U1065, Team2 Cell death, differentiation and cancer, 06000 Nice, France; 5 Université Nice Côte d’Azur, INSERM, U1065, Imaging platform, 06000 Nice, France; 6 CHU NICE, Département de Dermatologie, 06000 Nice, France

**Correction to: Mol Cancer (2021) 20:12**

**https://doi.org/10.1186/s12943-020-01306-2**

Following the publication of the original paper [1], the authors found out that the supplementary material is incomplete. Thus, the publication of correction to notify the readers on the error. Updated supplementary file is provided in this article.

## Supplementary Information

**Additional file 1.**
