# Supplementary figures and images for: ITGBL1 is a new immunomodulator that favors development of melanoma tumors by inhibiting natural killer cells cytotoxicity
Source: Mol Cancer. 2021 Jan 7;20:12. doi: 10.1186/s12943-020-01306-2 (PMC7789764; doi:10.1186/s12943-020-01306-2)

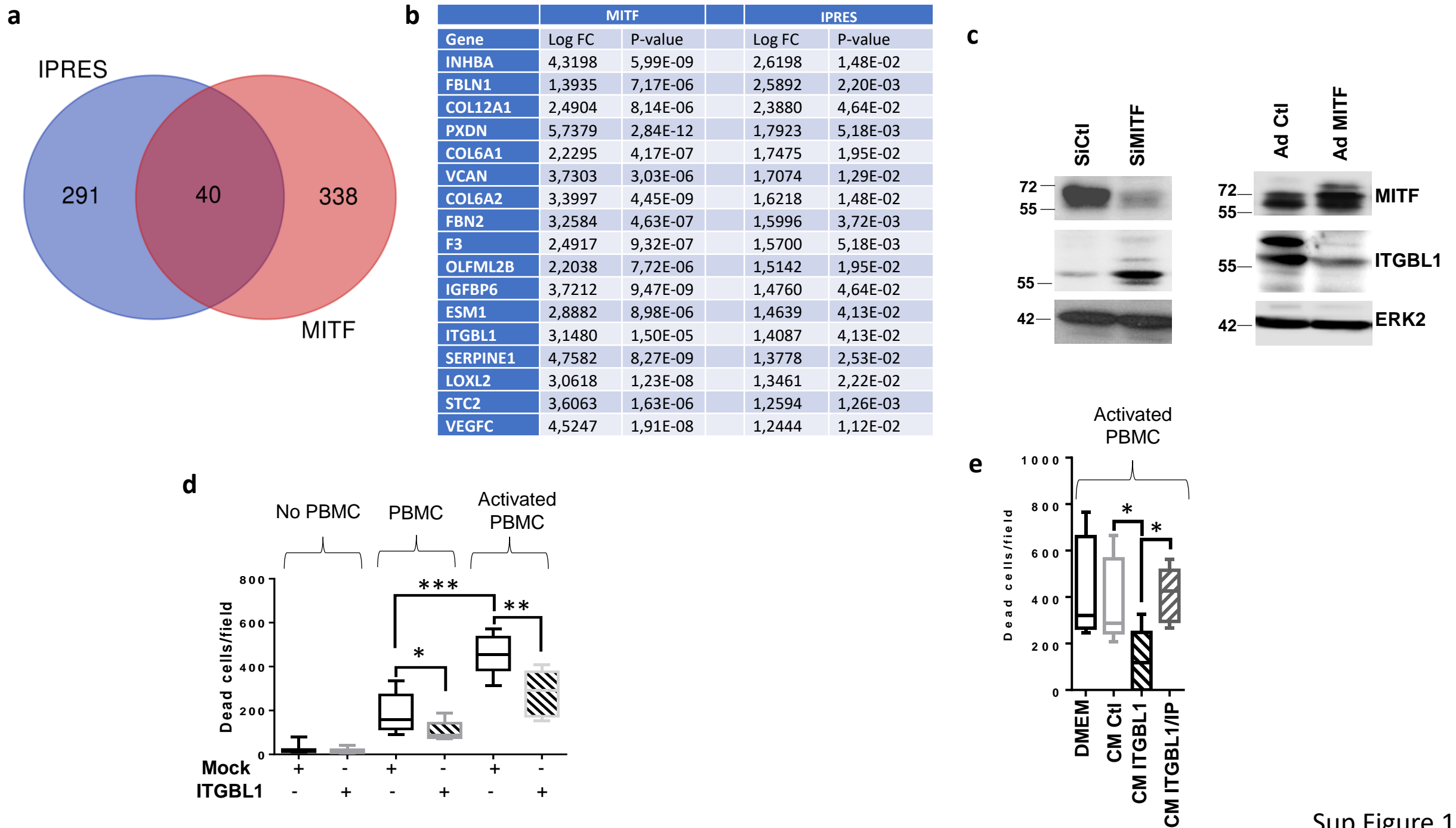

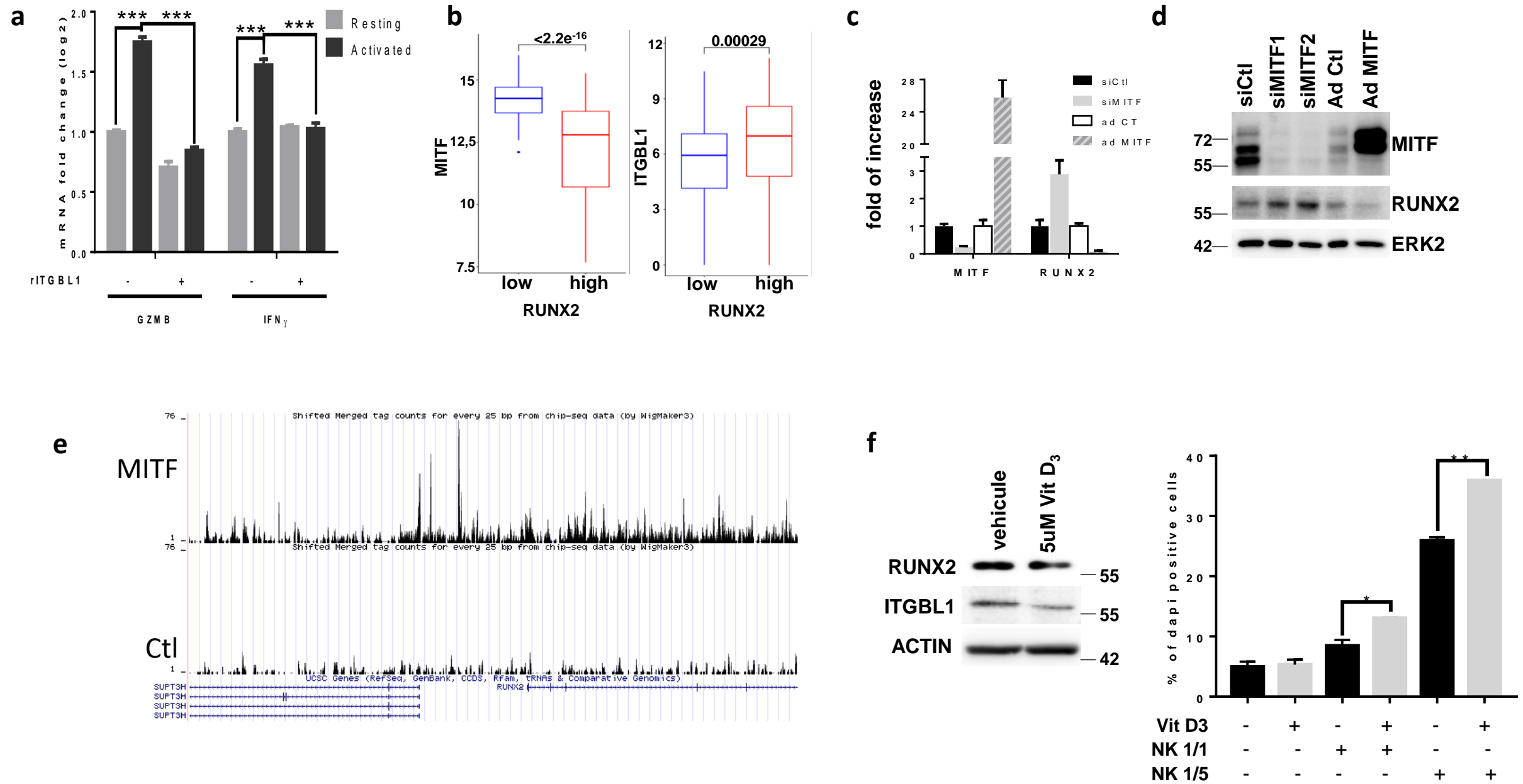

Sup figure 2

**a**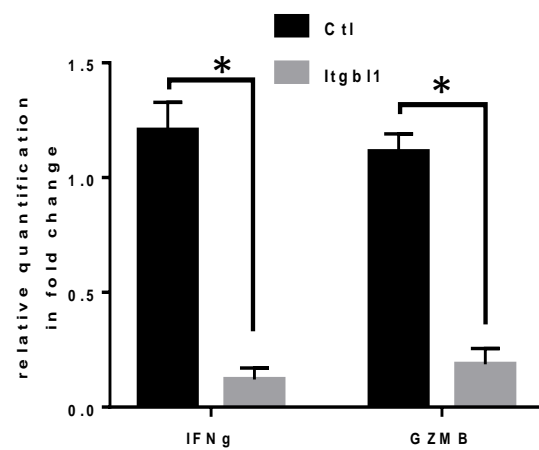**b**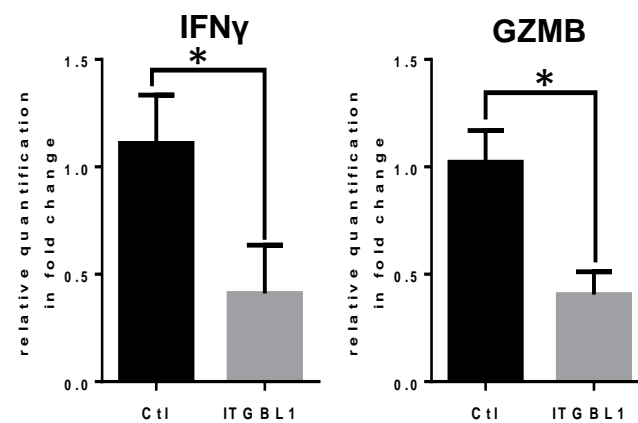

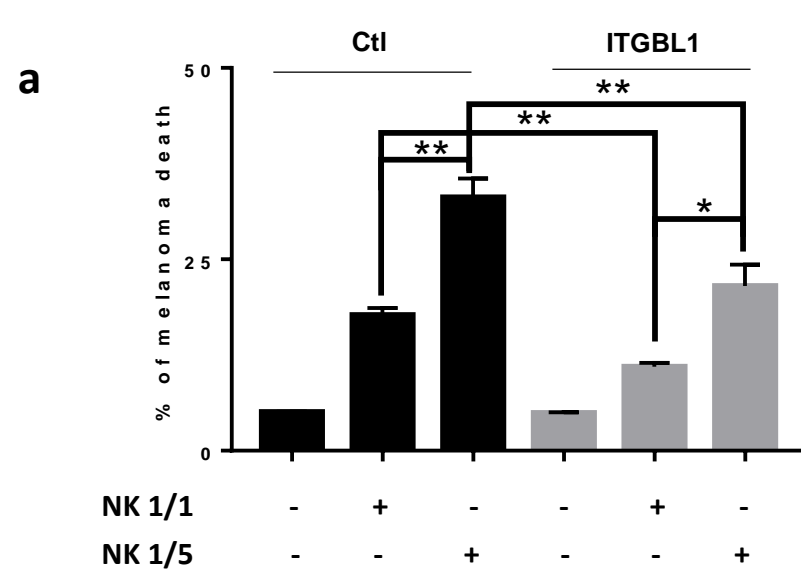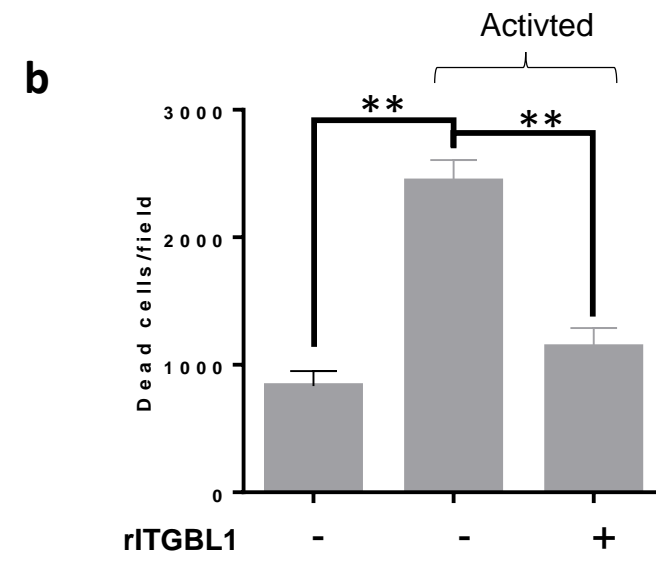

C-12-98

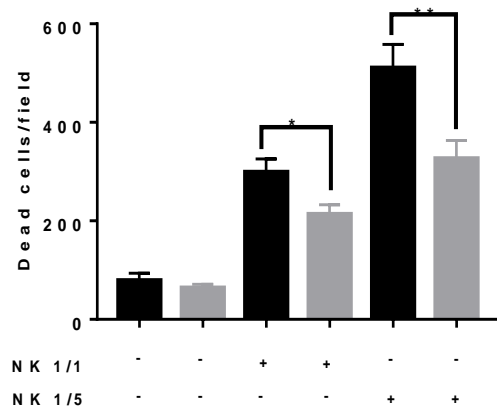

C-13-08

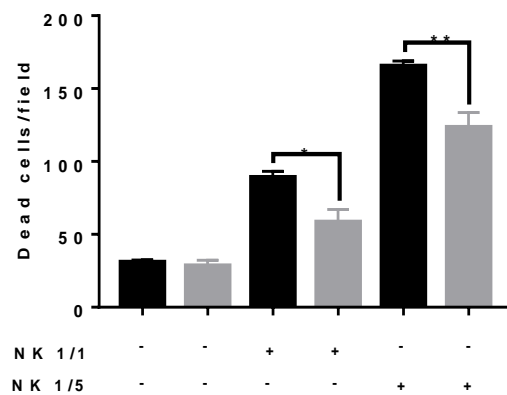

A375

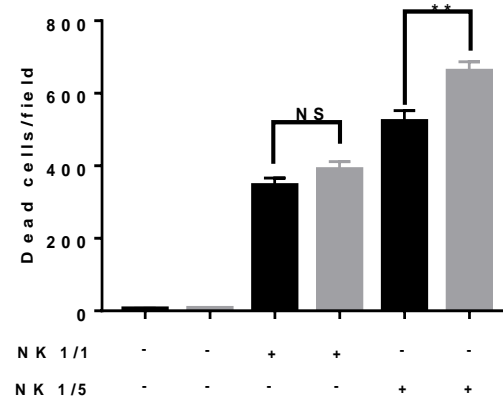

Hek293T

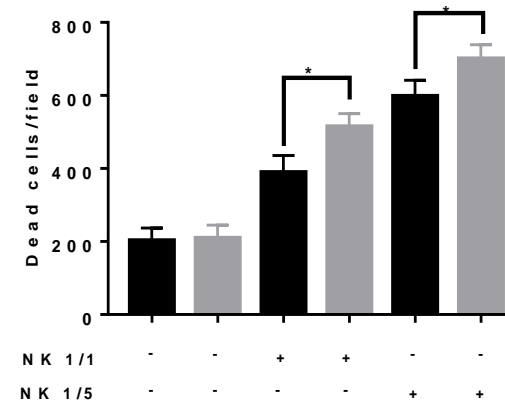

MHN

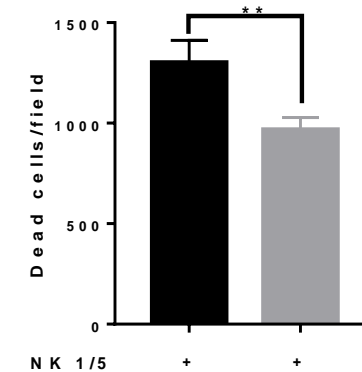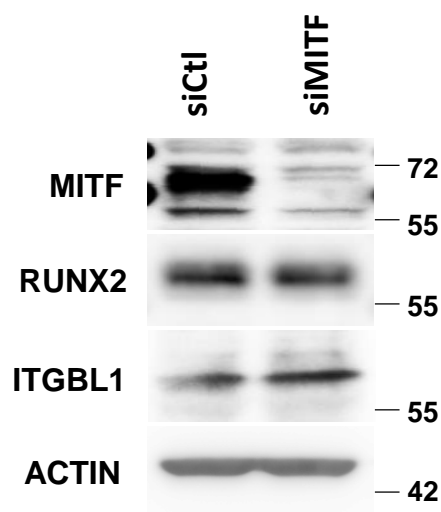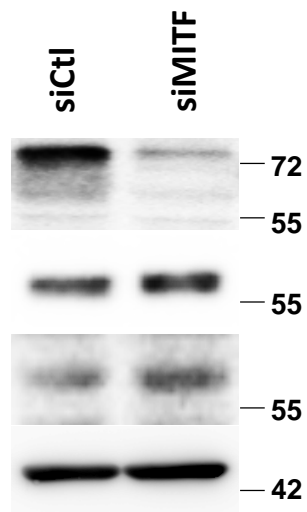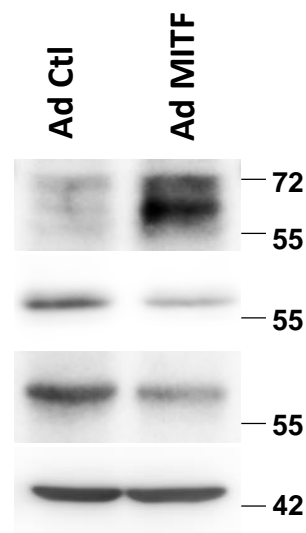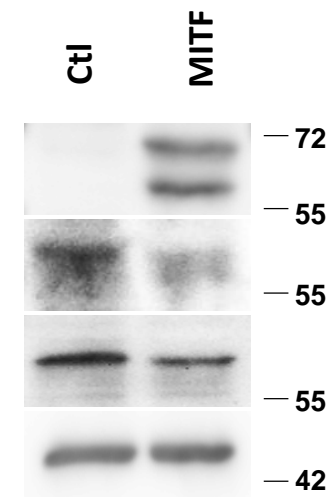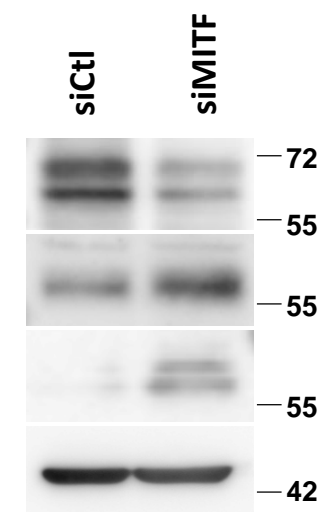

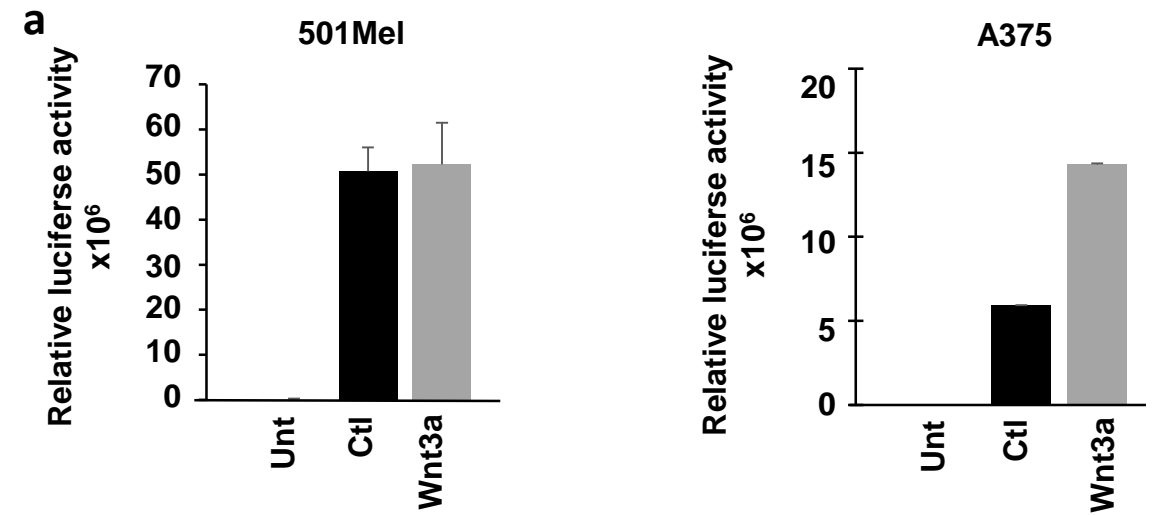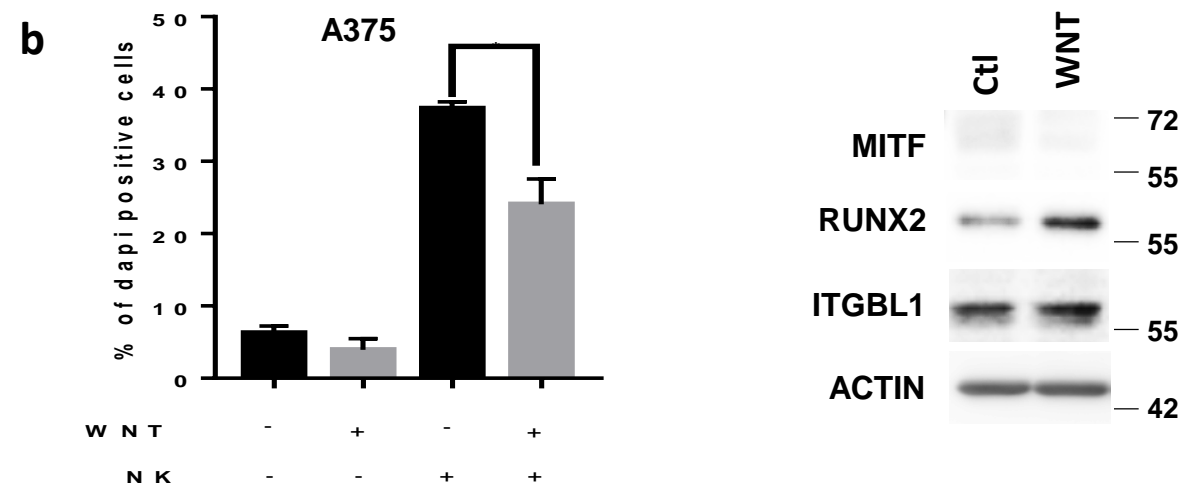

Supplement: Supplementary file 1 — Additional file 1. [file 12943_2020_1306_MOESM1_ESM.pdf]
