# Supplementary material for: Correction to: ITGBL1 is a new immunomodulator that favors development of melanoma tumors by inhibiting natural killer cells cytotoxicity
Source: Mol Cancer. 2021 Jan 27;20:21. doi: 10.1186/s12943-021-01319-5 (PMC7839296; doi:10.1186/s12943-021-01319-5)
Supplement: Supplementary file 1 — Additional file 1. [file 12943_2021_1319_MOESM1_ESM.pdf]

a

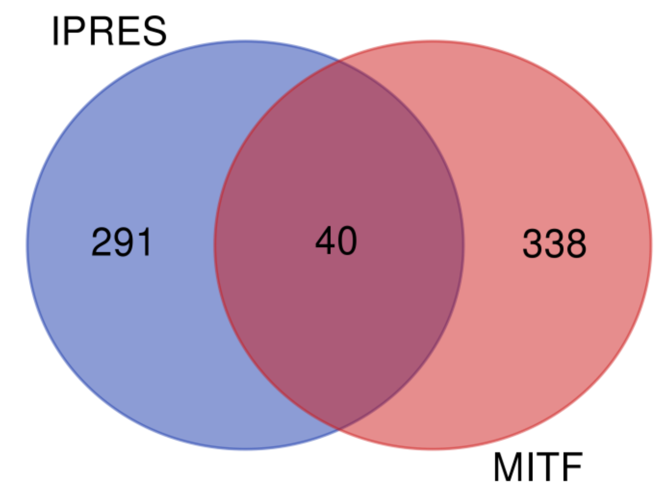

b

	MITF		IPRES	
Gene	Log FC	P-value	Log FC	P-value
INHBA	4,3198	5,99E-09	2,6198	1,48E-02
FBLN1	1,3935	7,17E-06	2,5892	2,20E-03
COL12A1	2,4904	8,14E-06	2,3880	4,64E-02
PXDN	5,7379	2,84E-12	1,7923	5,18E-03
COL6A1	2,2295	4,17E-07	1,7475	1,95E-02
VCAN	3,7303	3,03E-06	1,7074	1,29E-02
COL6A2	3,3997	4,45E-09	1,6218	1,48E-02
FBN2	3,2584	4,63E-07	1,5996	3,72E-03
F3	2,4917	9,32E-07	1,5700	5,18E-03
OLFML2B	2,2038	7,72E-06	1,5142	1,95E-02
IGFBP6	3,7212	9,47E-09	1,4760	4,64E-02
ESM1	2,8882	8,98E-06	1,4639	4,13E-02
ITGBL1	3,1480	1,50E-05	1,4087	4,13E-02
SERPINE1	4,7582	8,27E-09	1,3778	2,53E-02
LOXL2	3,0618	1,23E-08	1,3461	2,22E-02
STC2	3,6063	1,63E-06	1,2594	1,26E-03
VEGFC	4,5247	1,91E-08	1,2444	1,12E-02

c

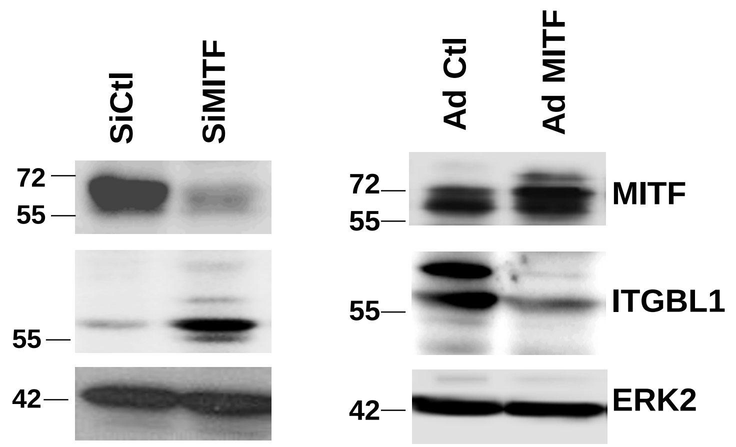

d

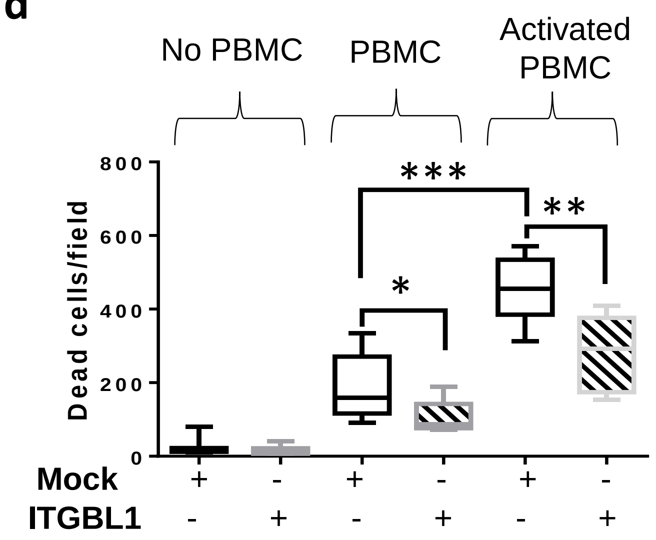

e

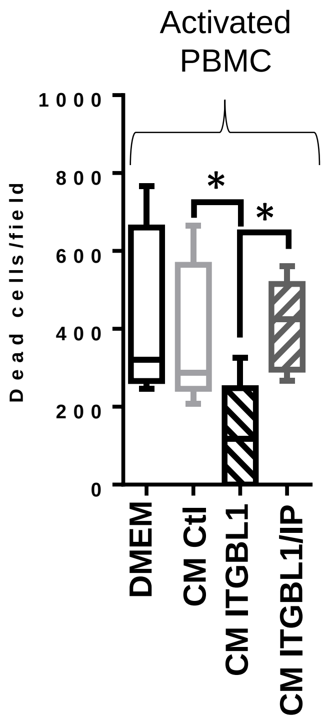

Sup Figure 1

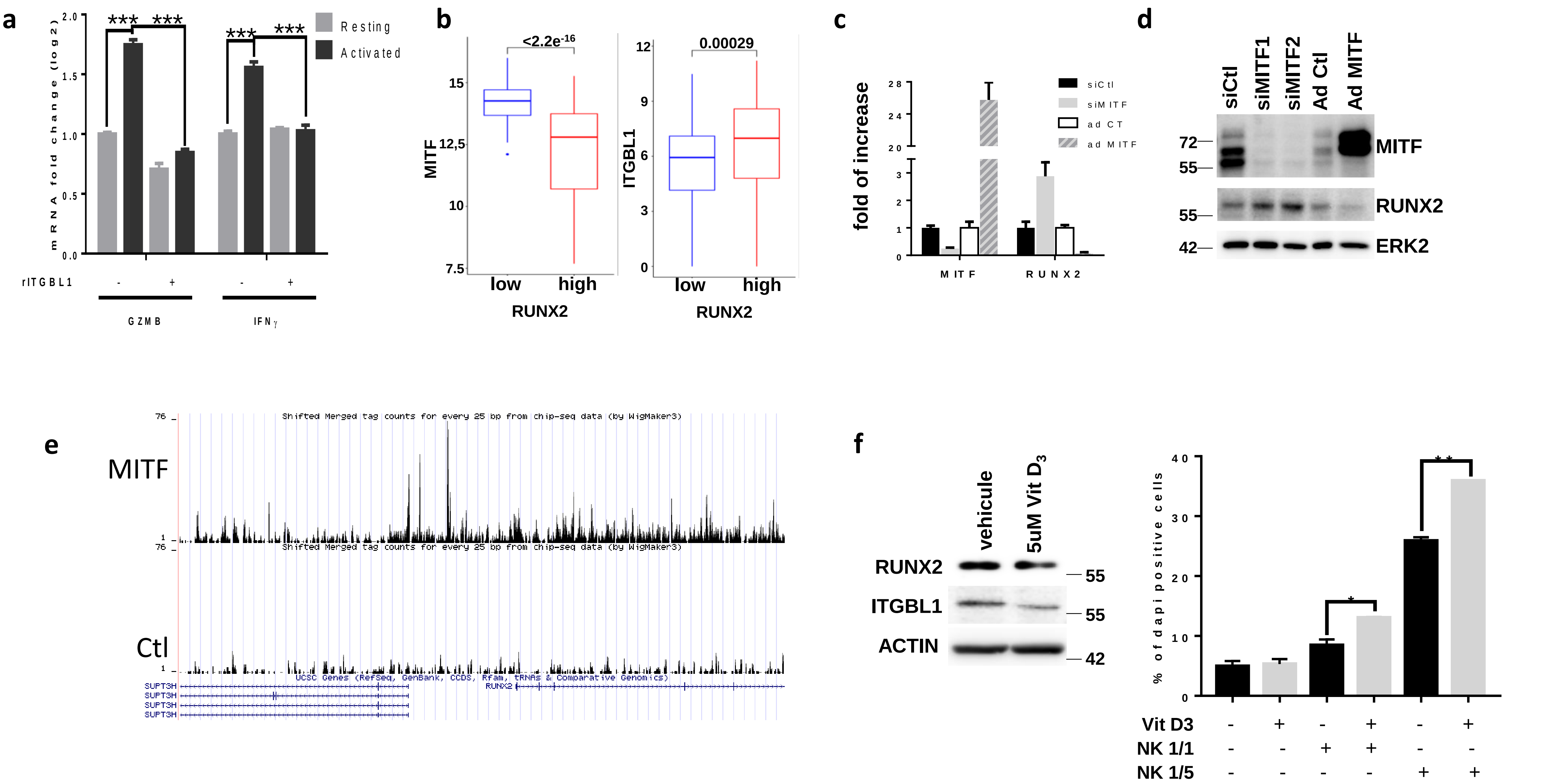
a
mRNA fold change (log2)
Resting
Activated
rITGBL1
GZMB
IFNγ
b
MITF
ITGBL1
<2.2e-16
0.00029
low
high
RUNX2
c
fold of increase
siCtl
siMITF
ad CT
ad MITF
MITF
RUNX2
d
siCtl
siMITF1
siMITF2
Ad Ctl
Ad MITF
MITF
RUNX2
ERK2
e
MITF
Ctl
Shifted Merged tag counts for every 25 bp from chip-seq data (by WigMaker3)
UCSC Genes (RefSeq, GenBank, CCDS, Rfam, tRNAs & Comparative Genomics)
SUPT3H
RUNX2
f
vehicule
5uM Vit D3
RUNX2
ITGBL1
ACTIN
% of dapi positive cells
Vit D3
NK 1/1
NK 1/5

Sup figure 2

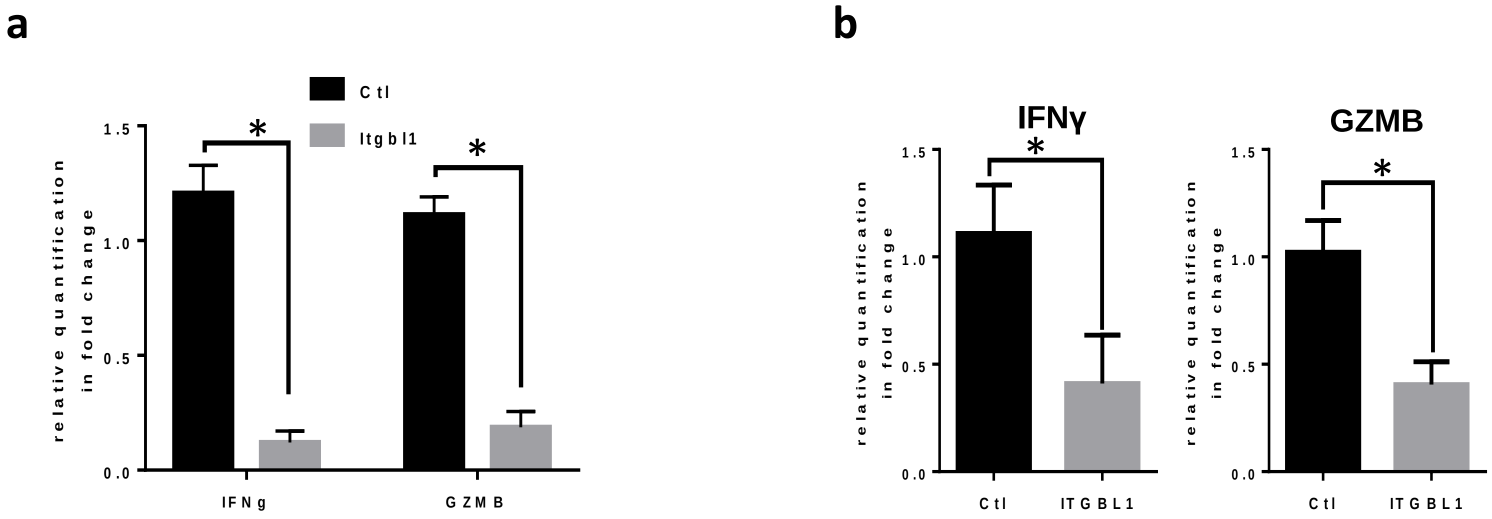
a
Ctl
Itgbl1
relative quantification in fold change
1.5
1.0
0.5
0.0
*
*
IFNg
GZMB
b
IFNγ
GZMB
relative quantification in fold change
1.5
1.0
0.5
0.0
*
*
Ctl
ITGBL1
Ctl
ITGBL1

Sup Figure 3

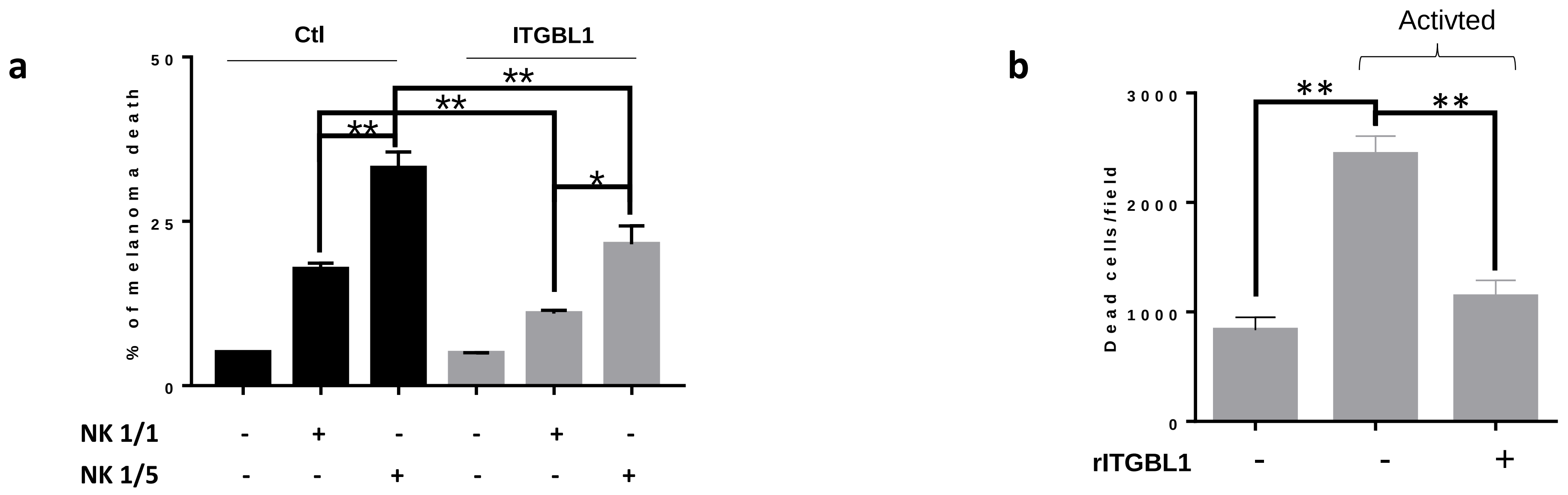

a
Ctl
ITGBL1
50
25
0
% of melanoma death
**
**
**
*
NK 1/1
NK 1/5
b
Activted
3000
2000
1000
0
Dead cells/field
**
**
rITGBL1

Sup Figure 4

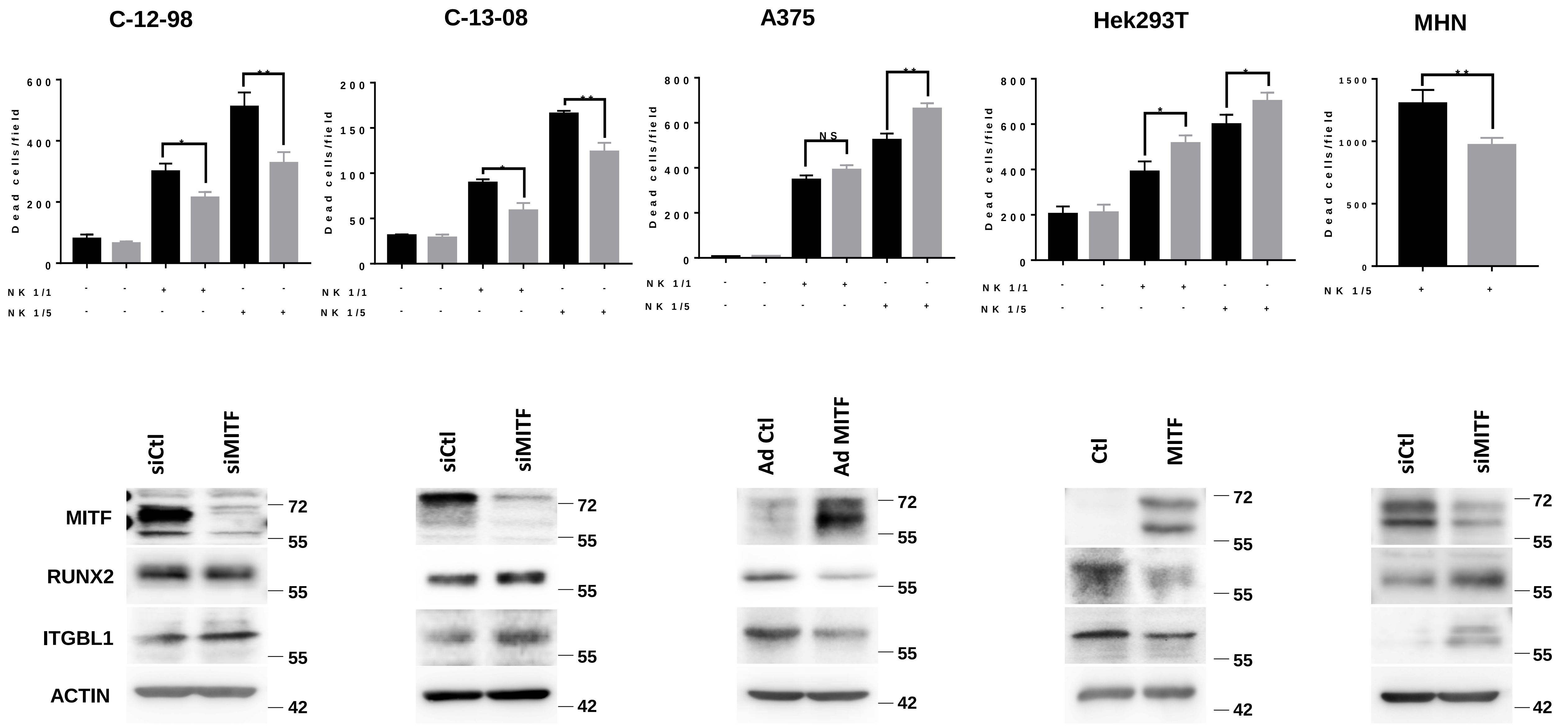
C-12-98
C-13-08
A375
Hek293T
MHN
Dead cells/field
NK 1/1
NK 1/5
NS
siCtl
siMITF
Ad Ctl
Ad MITF
Ctl
MITF
MITF
RUNX2
ITGBL1
ACTIN
72
55
42

Sup Figure 5

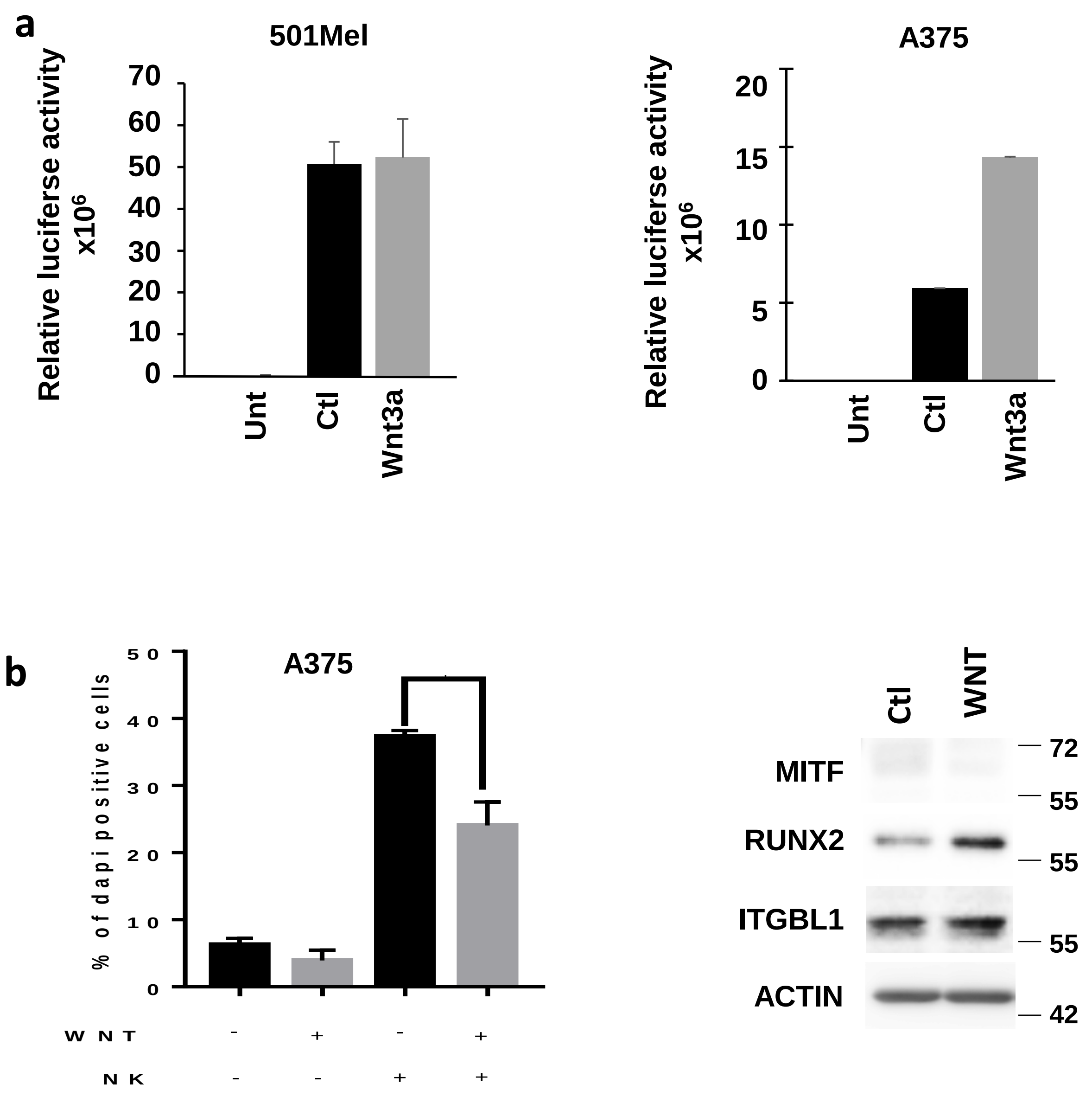
a
501Mel
Relative luciferse activity x10^6
70
60
50
40
30
20
10
0
Unt
Ctl
Wnt3a
A375
Relative luciferse activity x10^6
20
15
10
5
0
Unt
Ctl
Wnt3a
b
A375
% of dapi positive cells
50
40
30
20
10
0
WNT - + - +
NK - - + +
Ctl
WNT
MITF
RUNX2
ITGBL1
ACTIN
72
55
55
55
42

Sup Figure 6

Supplemental figure legend:

Sup figure1

a) Venn diagram of the transcriptomic analysis of genes that are upregulated in non-responders to anti-PD1 immunotherapies (IPRES signature) (blue) and genes upregulated in low-MITF melanoma cells (red) from CCLE Broad database. Overlapping regions are common genes upregulated between the both databases. **b**) Signature of top 17 genes encoding for secreted proteins in common between upregulated gene in IPRES and CCLE database. **c**) WM3912 were transfected with control or MITF siRNA (left panel) or infected with control or MITF adenovirus (right panel), then MITF and ITGBL1 expression was analyzed by western blot. ERK2 was used as loading control. **d**) ITGBL1 overexpressing SKMEL28 or parental with empty vector (P) cells were incubated for 48h with activated or quiescent PBMCs. Melanoma cell death was monitored with Incucyte. **e**) PBMCs were incubated for 48h in normal medium (DMEM), control conditioned media (CM Ctl) or SKMEL28-ITGBL1 conditioned media (CM-ITGBL1). When indicated ITGBL1 was first immune-depleted (CM-ITGBL1/IP) and then PBMCs were added to 501 melanoma cells and melanoma cell death was quantified 28h after and displayed as mean+/-SD of 3 independent experiments.

Sup figure2

a) Resting or activated PBMCs (with PMA/Iono) were stimulated with conditioned media from parental (Ctl) or ITGBL1 expressing SKMEL28 melanoma cells (ITGBL1), then mRNA was extracted and analyzed by QPCR for IFNγ, and Granzyme B expression. Results shown are mean+/-SD of 3 independent experiments. **b)** ITGBL1 (left panel) and MITF (right panel) expression analysis in high (red) and low (blue) Runx2 expressing tumors from the TCGA melanoma cohort. **c**) 501Mel melanoma cells were transfected with control or MITF siRNA or infected with control or MITF adenovirus. RNA was extracted and analyzed for MITF and RUNX2 mRNA expression by qPCR. **d**) Proteins from above 501Mel melanoma cells were probed for MITF and RUNX2 expression. ERK2 was used as a loading control. **e**) UCSC screenshots showing MITF occupancy over the RUNX2 loci (MITF). A GFP ChIP was performed as a negative control (Ctl). **f**) 501Mel melanoma cells were treated with 5 µM of VitD3 for 24h, then RUNX2 and MITF proteins analyzed by western blot. ACTIN was used as a loading control. On right panel, 501Mel melanoma pretreated with 5µM VitD3 (black) were incubated with NK cells at ratio 1/1 or 1/5. Cells were analyzed by FACS with dapi and melanoma death was quantified as % of melanoma positive dapi melanoma cells (mean+/-SD, n=3, **P*<0.05, ***P*<0.001).

Sup figure 3

a) mRNA from tumor overexpressing ITGBL1 (ITGBL1) or not (Ctl) in C57BL/6J were quantified for IFNγ and GZMB by QPCR and expressed as relative quantification in fold change+/-SD. **b**) mRNA from tumor overexpressing ITGBL1 (ITGBL1) or not (Ctl) in nude were quantified for IFNγ and GZMB by QPCR and expressed as relative quantification in fold change+/-SD.

Sup figure 4

a) Red stained melanoma cells (501Mel or Skmel-28) were cocultured with NK at different ratio (NK 1/1 or NK 1/5) for 24hours. Cells were analyzed by FACS with dapi and melanoma death was quantified as % of melanoma positive dapi melanoma cells (mean+/-SD, n=3, **P*<0.05, ***P*<0.001).* **b)** activated sorted CD56+ cells were incubated for 24h in presence or absence of recombinant ITGBL1 (5ng/ml). Cells were subsequently added to 501 melanoma cells and cell death was analyzed with Incucyte. Quantification of melanoma cell death is displayed as the mean+/-SD of 3 independent experiments.

Sup figure 5

Red stained melanoma cells (C-12-98, C-13-08, A375), HEK293T or NHEM were transfected with siRNA MITF (grey) or its control (black) or infected with adenovirus MITF (grey) or control (black) 48hours prior to be cocultured with NK cells at different ratio (NK 1/1 or NK 1/5) for 24hours. Melanoma cells death was analyzed with Incucyte. Quantification of melanoma cell death is displayed as the mean+/-SD of 3 independent experiments. On lower panel, proteins of transfected or infected cells were analyzed by western blot, then ITGBL1, RUNX2 and MITF proteins analyzed by western blot. ACTIN was used as loading control.

Sup figure 6

a) 501Mel or a375 were transfected with Topflash and pCMVβgal reporter and treated with 100ng/ml of Wnt3a, 5µM of PRI-724(PRI) or vehicle control (Ctl) for 24h. Non-transfected control was used for background. Luciferase and βgal were quantified and relative luciferase activity is displayed. **b)** Red stained melanoma cells (501Mel or a375) were pretreated for 24h with 100ng/ml of Wnt3A or 5µM of PRI-724 then cocultured with NK at different ratios (NK 1/1 or NK 1/5) for 24hours. Cells were analyzed by FACS with dapi and melanoma death was quantified as % of melanoma positive dapi melanoma cells (mean+/-SD, n=3, **P*<0.05, ***P*<0.001)*. On right panel, proteins of pretreated cells for 24hours were analyzed by western blot, then ITGBL1, RUNX2 and MITF proteins analyzed by western blot. ACTIN was used as loading control.

Methods

Experimental Models and Subject Details

Human study protocol was approved by the CPP ethics committees from Nice (DC-2015*2531). Informed consent was obtained from all subjects included in this study.

Mouse experiments were performed at the Animal Facilities of the Mediterranean Centre of Molecular Medicine (C3M) and the university of Liège according to French and Belgian laws. All mouse experiments at the C3M have been approved by Institutional Animal Care and the local ethical committee (APAFIS#202001281422585_v4).

Antibodies and reagents

Antibodies against ERK2 and HSP90 were from Santa Cruz Biotechnology. Anti FN1 antibodies was from BD Biosciences (Franklin Lakes, NJ, USA). Anti RUNX2 was from Cell Signaling Technology (Boston, MA, USA). MITF, IFNγ and Granzyme B antibodies were purchased from Abcam (Cambridge, MA, USA). ITGBL1 antibody was from Abgent (San Diego, CA, USA). Secondary antibodies conjugated to HRP were from Dako (Glostrup, Denmark) and alexa488 antibody and DAPI from Lifectech. BD cytofix/cytoperm plus kit was used for flow cytometry staining (San Jose, CA). Recombinant ITGBL1 was from My biosource (San Diego, CA). 1α,25-Dihydroxyvitamin D3 was purchased from Sigma-Aldrich (Lyon, France). For details, see resources table.

Cell culture

Cells (501Mel, WM3912, SKMEL-28 and WM-9, A375) were grown in Dulbecco's modified Eagle's (DMEM) medium (Invitrogen, Carlsbad, CA, USA) supplemented with 7% FCS and penicillin/streptomycin (100 U/ml/50 µg/ml) at 37°C and 5% CO2. Primary melanoma cells were grown in RPMI medium ((Invitrogen, Carlsbad, CA, USA) supplemented with 10% FCS and penicillin/streptomycin (100 U/ml/50 µg/ml) at 37°C and 5% CO2.

Cells were transfected with siRNA against MITF as previously described using lipofectamine RNAiMax. RUNX2 or a scrambled sequence shRNA were purchased from Sigma Aldrich. Viral particles were produced as described previously. For infections, 8×10^4 cells were seeded in six-well plates for 24 hrs, infected with viral supernatant for 48 hrs before processing. Cell infection with adenovirus expressing MITF (Vector Biolabs, Philadelphia, PA, USA) or the control adenovirus (empty vector) was carried out as described previously [23].
Luciferase assay was performed in 24-well dishes. Cells were transfected with 0.25 µg of Topflash plasmid and 0.025 µg of pCMVβGal to control transfection efficiency, using 2 µl of lipofectamine in a 200µl final volume. Luciferase assay was quantified using Luciferase assay system kit (Promega) according to manufacturer recommendations.

Peripheral Blood Mononuclear cells (PBMCs) preparation

PBMCs were obtained from healthy donors with informed consent following the Declaration of Helsinki according to the recommendations of an independent scientific review board. The project has been validated by The Etablissement Français du Sang. Blood samples were

collected using ethylene diamine tetra-acetic acid (EDTA)–containing tubes. Isolation of peripheral blood mononuclear cells (PBMCs) was performed by Ficoll gradient centrifugation (Lymphoprep®, Euromedex, France). Patient used for autologous experiment was undergoing anti PD-1 treatment.

In vivo experiments

The mice were maintained in a temperature-controlled facility (22°C) on a 12-hour light/dark cycle and were given free access to standard laboratory chow (UAR, Epinay-S/Orge, France). B16F10 or BP mouse melanoma cells were injected subcutaneously ($0.15X10^6$) into the flank of C57BL/6J or nude or NSG mice. Body weight and tumor volume were measured three times a week. Tumor size was measured with linear calipers and calculated using the formula: tumor width × tumor $length^2$×0.5. Mice were sacrificed after 15 days maximum by cervical dislocation. For immunophenotyping, tumors were dissociated in DMEM with 3mg/ml of collagenase A for 15 mins at 37°c, tumor suspension was filtered using a 70µm filter and stained for specifics immunes markers in staining buffer (PBS/2mM EDTA/ 0.5% BSA) on ice for 10 min according to manufacturer's recommendations. Frozen tumors were crushed and resuspended in Trizol. RNA purification from tumor was performed using Direct-zol RNA miniprep kit according to manufacturer recommendation, then QPCR was performed as described previously [24].

Western blot

Cells were solubilized for 10 min at 4 °C in buffer containing 50 mM Tris pH 8, 150 mM NaCl, 1% Triton X-100 and EDTA-free protease inhibitor cocktail (Roche Applied Science). Thirty micrograms of protein were separated by electrophoresis on 10% polyacrylamide SDS–polyacrylamide gel electrophoresis and transferred onto a polyvinylidene difluoride membrane (Immobilon, Millipore, Molsheim, France). The membrane was saturated for 1 hr at 25 °C in 10 mM Tris HCl, pH 7.4, 150 mM NaCl, 1 mM EDTA, 0.1% Tween 20, 3% bovine serum albumin (weight/volume), 5% gelatin (weight/volume) and incubated with primary antibodies overnight at 4 °C. After three washes of 5 mins in 10 mM Tris pH 7.4, 150 mM NaCl, 1% Triton X-100, the secondary antibody coupled with horse radish peroxidase (Dakopatts, Glostrup, Denmark) was applied and left for 1 hr at room temperature. After three additional washes, proteins of interest were revealed by ECL (Amersham, Uppsala, Sweden) using ImageQuant LAS-4000 Fujifilm (GE healthcare, Pittsburgh, PA).

ELISA

Supernatant from stimulated PBMCs were collected and IFNγ production was quantified by commercially available ELISA Kits (Peprotech, NJ, USA). Quantification was performed according to manufactured recommendations. Supernatant from SKMel-28 control or ITGBL1 overexpressing was analyzed using Elisa kit from My biosource (San Diego, CA) after 48 hrs in contact with cells according to manufacturer recommendations. Immunodepleted medium was obtained by adding 10 µg of anti-ITGBL1 antibody *per* ml of medium overnight at 4°C, then protein A/G immunomagnetic beads were added for 1 hr at 4°C before the tube was placed on the magnetic separator to remove the immunomagnetic beads-antibody complex.

Live imaging of melanocyte viability

Melanoma cells were plated in 96 well plates at 6000 cells *per* well. The next day, cells were stained with 0.5 µM CellTracker® Red CMPTX dye for 20 mins at 37° C according to manufacturer's recommendations (Molecular Probes, USA) before adding either 100,000 cells *per* well of unstimulated PBMCs, the same number of activated PBMCs (pre-treated for 48 hrs with 10ng/µl of PMA (Sigma Aldrich) and 10ng/µl of ionomycin (Sigma Aldrich)) or no PBMCs at all. Incucyte® green Cytotox Reagent (100 nM, Essen Bioscience, Michigan, USA) was added to all wells prior imaging and melanoma cell death was monitored in real-time using IncuCyte® Zoom live-cell imaging system (Essen Biosciences). Cells were maintained at 37° C in humidified environment and 5% CO_2 scanning every 2 hrs at 10X magnification. Multiple images were collected *per* well and quantification of dead melanoma cells (yellow co-localised cells) was analysed using the integrated Zoom® software. In experiments with conditioned media, PBMCs were preincubated in their respective media for 48 hrs with PMA/ionomycin prior to their addition to melanoma cells. Vitamin D3 (5 µM) was added to melanoma cells for 48 hrs seeded in 96-well plates then media was replaced before adding PBMCs.

NK cells activity against melanoma

Melanoma cells were stained with 0.5 µM CellTracker® Red CMPTX dye for 15 minutes at 37°C according to manufacturer recommendations (Molecular Probes, USA) before being plated in 48 well plates at density of 25000 cells *per* well. Five hours later, NK-92 cells were added at a ratio of 1:1 with melanoma cells and then a ratio of 1:5 (that is 5 times more NK than melanoma cells). Cells were then incubated for 24h at 37°C in humidified environment and 5%CO_2. In case of treatment with anti-PD1, NK cells were incubated for 30 mins with 5µg/ml of anti-PD1 or its isotype control prior to be added to melanoma cells. 24hours later, cells were detached and analysed by flow cytometry for melanoma death. Briefly, cells were resuspended in 300µl of PBS 0.5% BSA, 2mM EDTA. Dapi was added to final concentration of 1µg/ml just before analysis using MACsQuant Cytometer (Miltenyi Biotech, Paris FRANCE).

Bioinformatic analysis

We used the Skin Cutaneous Melanoma (SKCM) dataset from The Cancer Genome Atlas (TCGA). RNA-seq and clinical data were downloaded from the TCGA data portal (https://portal.gdc.cancer.gov). RNA-seq data were normalized using the Bioconductor package DESeq2 and log2 transformed. For each gene, we ranked patients by their ITGBL1 expression level, and we classified the 25% highest and the 25% lowest in the two groups. We investigated the cellular components of the tumor microenvironment by inferring relative fractions of different immune cell types using CIBERSORT [25] from both groups of patients based on the ITGBL1 expression profile (https://cibersort.stanford.edu/). P-values were calculated using the Wilcoxon rank sum test. Survival curve from TCGA was obtained using R survival package. Overall survival was performed using primary tumors data from TCGA. Significance was assessed using the log-rank test.

RESOURCES TABLE

REAGENT or RESOURCE	SOURCE	IDENTIFIER
**Antibodies**		
Anti RUNX2 (D1L7F)	Cell signaling	# 12556S
Anti ERK2	Sant Cruz Biotech.	sc-1647
Anti HSP90	Sant Cruz Biotech.	Sc-13119
Anti MITF (C5)	Abcam	ab12039
Anti IFNγ	Abcam	Ab9657
Anti ITGBL1 center	Abgent	# AP8781c
Anti mouse Alexa488 Labeled	Lifetech	A28175
Anti mouse and anti Rabbit HRP	Dako	P0447 and P0448
Anti mouse PD-1	BioXCell	BE0146
Mouse IgG1 control	BioXCell	BE0083
Rat IgG2 control	BioXCell	BE0089
**Chemicals, Peptides, and Recombinant Proteins**		
1α,25-Dihydroxyvitamin D3	Sigma Aldrich	D1530
Ionomycin	Sigma Aldrich	I0634
Phorbol 12-myristate 13-acetate	Sigma Aldrich	P1585
Recombinant ITGBL1 from elisa Kit	My biosource	MBS-9328278-96
CellTracker® Red CMPTX	Lifetech	C34552
Bio-Adembeads Protein AG, 250 IPP	Ademtech	04631
Lymphoprep	Eurobio Lifesciences	CMSMSL01-01
**Critical Commercial Assays**		
Human Integrin beta-like protein 1, ITGBL1 ELISA KIT	My biosource	MBS-9328278-96
Human IFNG ELISA KIT	Peprotech	900-K27

Human recombinant WNT3A	R&D Systems	5036-wn-010
PRI-724	Cliniscience	A15856
**Experimental Models: Cell Lines**		
501Mel	Ludwig Institute for Cancer Research	Gift form Dr Goding Colin
SKMEL-28	ATCC	HTB-72
WM3912	The Wistar Institute	Gift form Dr Meenhard Herlyn
WM-9	The Wistar Institute	Gift form Dr Meenhard Herlyn
B16-F10	ATCC	CRL-6475
A375	ATCC	CRL-1619
HEK293T	ATCC	CRL-11268
C-12-98	Patient donor	Internal
C-13-08	Patient donor	Internal
NHEM	Healthy donor	internal
**Experimental Models: Organisms/Strains**		
Nude	Janvier Labs	Rj:ATHYM-Foxn1nu/nu
C57BL/6J	University of Liège	Internal breeding
C57BL/6J	Janvier Labs	C57BL/6J
NSG	C3M	Internal breeding
**Oligonucleotides**		
L32 Forward	Lifetech	TGTCCTGAATGTG GTCACCTGA
L32 reverse	Lifetech	CTGCAGTCTCCTTG CACACCT

ITGBL1 Forward	Lifetech	ACCATCCCTGGTC ACACTTG
ITGBL1 Reverse	Lifetech	CCTGTGTGAGTGC CATGAGT
IFNG Forward	Lifetech	TCGGTAACTGACTT GAATGTCCA
IFNG Reverse	Lifetech	TCGCTTCCCTGTTT TAGCTGC
GZMB Forward	Lifetech	CCCTGGGAAAACA CTCACACA
GZMB Reverse	Lifetech	GCACAACTCAATG GTACTGTCG
Mouse L32 Forward	Lifetech	TTAAGCGAAACTG GCGGAAAC
Mouse L32 reverse	Lifetech	TTGTTGCTCCCATA ACCGATG
Mouse IFNG Forward	Lifetech	ACAGCAAGGCGAA AAAGGATG
Mouse IFNG Reverse	Lifetech	ACAGCAAGGCGAA AAAGGATG
Mouse GZMB Forward	Lifetech	CCACTCTCGACCC TACATGG
Mouse GZMB Reverse	Lifetech	GGCCCCCAAAGTG ACATTTATT
siRNA MITF #1	Lifetech	GGUGAAUCGGAUC AUCAAG
siRNA MITF #2	Lifetech	AGCAGUACCUUUC UACCAC
siRNA Control	Lifetech	UUCUCCGAACGUG UCACGU
**Recombinant DNA**		
Human ITGBL1 ORF expression clone	Origene	RC207781
RUNX2 MISSION shRNA	Sigma Aldrich	SHCLNG-NM_004348

Human adenovirus MITF	Vector Biolabs	AAV-215489
Human adenovirus GFP	Vector Biolabs	AAV2-GFP
Topflash plasmid	Addgene	#12456
**Software and Algorithms**		
TCGA data portal	NIH	https://portal.gdc.cancer.gov
CIBERSORT	Stanford	https://cibersort.stanford.edu/
Bioconductor package DESeq2	Michael Love, Simon Anders, Wolfgang Huber	https://bioconductor.org/packages/release/bioc/html/DESeq2.html
Package survival	Terry M Therneau	https://cran.r-project.org/web/packages/survival/index.html
MacsQuant	Milteniy	https://www.miltenyibiotec.com
IncuCyte software	Essen Biosciences	www.essenbioscience.com/
R project R 3.4.2	R-project	https://www.r-project.org/
PRISM 6	GraphPad	https://www.graphpad.com/
